# An Adaptive Network Design for Advanced Metering Infrastructure in a Smart Grid

**DOI:** 10.3390/s22228625

**Published:** 2022-11-09

**Authors:** Jin-Woo Kim, Jaehee Kim, Jaeho Lee

**Affiliations:** 1 Department of Statistics, Duksung Women’s University, Seoul 01369, Korea; 2 Department of Software, Duksung Women’s University, Seoul 01369, Korea

**Keywords:** IEEE 802.15.4, MAC, smart grid, IoT, CSMA/CA

## Abstract

A smart grid is a next-generation intelligent power grid that can maximize energy efficiency by monitoring power information in real time and by controlling the flow of power by introducing IT communication technology to the existing power grid. In order to apply a wireless communication network to a smart grid, it is necessary to be able to efficiently process large amounts of power-related data while enabling a high level of reliability and quality of service (QoS) support. In addition, international standards-based design is essential considering compatibility and scalability. The IEEE 802.15.4 standard is considered to be the most powerful communication method for processing data through the smart grid AMI. To reduce the energy consumption, as the duty cycle of the superframe increases, the probability of the congestion increases. However, this binary exponential algorithm in IEEE 802.15.4 standard does not account for the application of traffic characteristics that essentially negatively affect the smart grid network performances in terms of packet delivery ratio and time delay. Therefore, in this paper, we propose a new transmission scheme to reduce performance degradation by excessive collisions in the content access period (CAP), when data transmission is performed in IEEE 802.15.4 applied to smart grids. In addition, we investigated the main research topics required when applying wireless networking technology to smart grids and suggested improvement measures. Simulation results showed that the proposed scheme increased the data delivery rate and reduced the latency, and it was confirmed that reliability was improved.

## 1. Introduction

A smart grid is a next-generation power system and management system that combines information and communication technology with existing power grids. When power is used, the smart grid supports real-time power information exchange between suppliers and consumers, energy conservation, and renewable energy supply. The smart grid consists of smart meter-based advanced metering infrastructure (AMI), monitoring technology, integration of information and communication technology, and distribution network management.

Smart grid communication systems require high-performance data delivery that enables reliable remote control systems with the ability to monitor real-time operating conditions and performance of power systems. However, communication systems currently used in power systems are slow, isolated from each other, and cannot support the comprehensive communication required by smart grids. Therefore, there is a need for an open communication structure that fully supports standards-based interoperability.

The general communication network structure used in smart grids can be divided into three parts. The first is the home area network (HAN), which consists of a communication infrastructure for connection and information exchange between sensors and devices inside a house. Second, there is the neighborhood area network (NAN), which consists of a number of data collectors connected to smart meters that collect and distribute information inside the house. Finally, there is a wide area network (WAN) that connects data collectors that collect information from the NAN and the utility control center of the power company to transmit information [1,2].

In such a smart grid network environment composed of various large and small communication networks, the AMI efficiently delivers instrumentation data information related to power generation and consumption through efficient two-way communication between the power provider (utility) and the smart meters constituting the HAN. In addition, AMI provides a network infrastructure to effectively communicate power control and management information to balance supply and demand of power, including real-time power price information and power outage risk information [3,4].

In particular, the Intelligent Project, which has been under the leadership of the United States Department of Energy (DOE) since 2003, aims to build intelligent future power infrastructure technologies with high quality and high reliability. Here, the characteristics of the needs that the communication network must satisfy are defined as follows [5,6,7].

First, all networks used in the smart grid should be based on standardized protocols. This means that in environments where different types of equipment are used, from low-performance simple modules to high-performance complex systems, integration must be possible with minimal change and compatible with universal industrial standards. Second, it should be possible to support low transmission delay and real-time two-way communication to implement basic functions of the smart grid such as distributed automation and real-time power control. Third, scalability and mobility must be ensured. In a large-scale infrastructure structure such as a smart grid, there are numerous input/output units, and these input/output units should be easily added and expanded according to system changes. It should also be possible to support mobility to reflect the characteristics of the wireless network as much as possible for the mobile equipment present in the system.

A wireless sensor network (WSN) can be cited as a wireless network protocol that meets these requirements, which is expected to enable proper inspection and improvement of power systems with versatile and promising innovations [8].

Currently, a common wireless personal area network (WPAN) protocol used in smart grid AMI communication is IEEE 802.15.4, the wireless standard of IEEE [9,10,11]. However, just as ensuring minimal reliability is essential in industrial networks, quality of service (QoS) including network reliability and stable transmission characteristics must be considered for integration of systems using wireless networks.

In particular, in order to use IEEE 802.15.4 as a control network for the smart grid, it is essential to improve QoS that can improve transmission delay characteristics. This is because due to the characteristics of the carrier sensing multiple access with collision (CSMA/CA) technique used as a medium access control (MAC) method, the deviation of the transmission delay increases due to the content between nodes, and random transmission delay can damage the stability of the system applying the network. 

Because IEEE 802.15.4 basically uses CSMA/CA competition for media access approaches, it is difficult to provide QoS, as the number of wireless nodes to be transmitted increases the probability of collision, and the network performance decreases as the number of data retransmissions increases. In this paper, we propose an improved MAC protocol that can satisfy the various requirements when developing AMI applications in large AMI network environments. The main contributions of this paper are as follows.

- The proposed scheme improves the performance of wireless network protocol for smart grid applications. In particular, we propose an improved MAC protocol for wireless network to provide QoS requirements for smart grid applications. Since the proposed scheme sets the backoff interval of the devices differently, congestion and delay can be reduced even if multiple devices attempt to transmit.
- The performance of the proposed scheme is compared with the well-known QoS support scheme proposed in the literature. In particular, we study the performance of the proposed scheme in terms of end-to-end delay, energy consumption, and packet delivery ratio.
- Finally, to show the advantages of the proposed protocol, we applied the proposed protocol to a real smart grid application. We prove that the proposed protocol shows satisfactory performance by applying the defined traffic in the smart grid to the simulation.

## 2. Background

### 2.1. An Overview of IEEE 802.15.4 Standard

**Figure 1:**
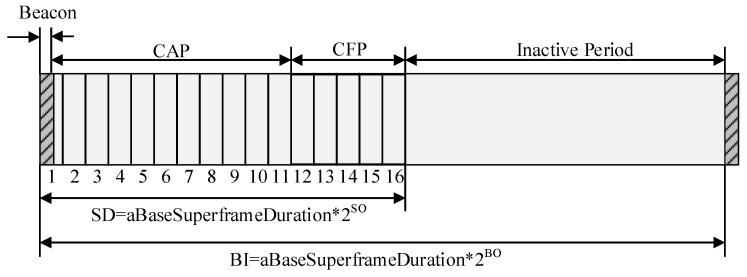
The superframe structure in the IEEE 802.15.4 standard.

Figure 1 shows the superframe structure in IEEE 802.15.4 standard.

The coordinator in the personal area network (PAN) can selectively restrict the channel time using a superframe structure as shown in Figure 1. In Figure 1, the superframe structure is determined by a beacon transmitted by a coordinator and is divided into 16 slots with the same length. The beacon frame is transmitted in the first slot of the superframe, and the beacon interval can be from a minimum of 15 ms to a maximum of 245 s. Beacons are used to synchronize devices connected in the PAN, identify the PAN, and describe the superframe structure. The time between two beacon frames is divided into 16 equal time slots regardless of the superframe period. The device can send data at any time in the time slot but must complete the data transmission/reception before the next super frame period. The beacon frame includes a superframe specification, a current notification of pending node message, etc.

The superframe can be divided into an active period and an inactive period. The inactive period is used as a low power mode. The active period can be divided into a contention access period (CAP) and a contention free period (CFP). Devices that try to transmit data frames in the CAP access the wireless channel using a slotted CSMA/CA mechanism. On the other hand, the CFP is divided into guaranteed time slots (GTS) and is used to transmit data frames to be guaranteed QoS. For example, a coordinator can provide a GTS, mainly for real-time applications or for applications requiring special bandwidth. The PAN coordinator can allocate up to seven GTSs, and a GTS consists of one or more time slots. The inactive period allows the coordinator to enter a low power mode without interacting with the PAN.

The superframe structure can be adjusted using superframe order (SO) and beacon order (BO) values contained in the beacon frame (0 ≤ SO ≤ BO ≤ 14). If both SO and BO have a value of 15, then the PAN operates as a non-beacon-enabled mode and does not have the superframe structure. The superframe is divided into superframe duration (SD) interval and beacon interval (BI) by BO value and SO value, respectively. The SD interval, that is, the active period, is always divided into 16 slots regardless of the BI value, and this interval is again divided into CAP and CFP intervals. The beacon frame includes the CAP, the CFP, and the GTS allocation information, and devices in the PAN update information for the superframe structure using the beacon frame.

To reduce the energy consumption, the coordinator can reduce the duty cycle of the PAN by reducing the SD length. However, if the length of the SD decreases, and the inactive period increases, the number of devices that do not transmit data frames in the SD increases. In addition, the number of devices that try to transmit data frames increases in the active period of the next superframe. Therefore, as the duty cycle of the superframe increases, the probability of the congestion increases. To address this problem, IEEE 802.15.4 standard provides the random backoff scheme for the packet transmission. However, if the concentration of traffic becomes severe in the next superframe, it is difficult to solve the problem with the backoff scheme. In addition, if a packet transmission attempt is rapidly increased in the next active period, packet transmission attempts occur at the same time in the CAP, and the congestion occurs in the CAP.

### 2.2. QoS Limitations in IEEE 802.15.4 Standards

**Figure 2:**
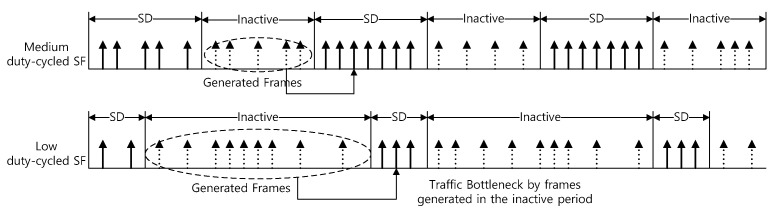
An example of data transmission in IEEE 802.15.4 standard.

This section describes the channel approach and QoS limitations of IEEE 802.15.4. The IEEE 802.15.4 MAC identifies the state of the channel before transmitting data from any wireless node, and the node uses a random backoff algorithm to avoid collisions. That is, a random backoff time is selected, and a channel is detected while waiting until the backoff time reaches 0. Figure 2 shows a data transmission process when the IEEE 802.15.4 WPAN is operated with a low duty cycle. As shown in Figure 2, the lower the duty cycle, the more devices receive data frames from the higher layer during the inactive period, and they attempt to transmit these data frames at the beginning of the next superframe. In this case, the devices come into contention with a fixed initial backoff setting for the packet transmission. However, if the concentration of traffic becomes severe, this problem cannot be solved by the backoff mechanism alone. In addition, if a packet transmission attempt increases rapidly in the next superframe duration (SD), a packet transmission attempt is concentrated in a contention access period (CAP), which causes a bottleneck phenomenon.

### 2.3. Related Works

Recently, studies are being conducted to incorporate IoT into smart grids [12,13,14,15,16,17,18,19,20,21]. However, they are focused on developing web services using IoT. Our interest is not in the development of web services, but in establishing the communication infrastructure that enables them.

The use of WSNs for the monitoring purposes in smart grids has been studied in the literature. The challenges and opportunities of using WSNs in smart grid application are discussed extensively in [22]. In [23], the authors conducted research on various communication technologies used in power systems. They reviewed smart grid communication methods based on the IEEE 802.15.4 standard and how large amounts of real-time data collected from AMI can be exchanged effectively, efficiently, inexpensively and securely. In [24], the authors proposed a lightweight mesh networking solution to reduce the cost of deploying WSN core networks while maintaining the same level of energy consumption for sensor nodes. They developed a prototype system in the laboratory to demonstrate the feasibility of the proposed mesh solution using the BLE chipset. In [25], the authors proposed a system for sensor network applications for smart grid transmission, transformation, distribution, and power consumption through an in-depth analysis of the application scenarios of wireless sensor networks in smart grids. In [26], the authors proposed a cross-layer design that supports energy efficiency. The authors proposed a variable-length TDMA scheme in which slot length is optimally allocated according to routing requirements while minimizing energy consumption across the network. In [27], the authors proposed a unified XLM (cross-layer module) that achieves efficient and reliable event communication in WSNs with minimum energy expenditure. In [28], the authors presented a WSN-based intelligent lighting control system that considers user activities and profiles. In the proposed system, the wireless sensor is responsible for measuring current lighting and models the lighting requirements of the user, taking into account the user’s activity. The authors proposed lighting decision algorithms and device control algorithms to meet user requirements and save energy. In [29], the authors proposed a WSN system to monitor partial-discharge (PD) activity in high-voltage transformers. The proposed scheme in [29] collects PD data from individual sensor nodes and transmits collected data to the gateway. In [30], the authors proposed a Time of Use (TOU)-aware energy management scheme to reduce the peak load of smart grid applications based on WSNs. In [31], the authors proposed XLP (Cross-Layer Protocol) to realize efficient and reliable communication in WSNs. XLP provides the functionalities of medium access, routing, and congestion control. In [32], the authors proposed the IEEE 802.15.4 protocol with enhanced QoS support to support stable networking and communication of power distribution systems. The authors present a Markov chain-based mathematical model developed to model Zigbee’s MAC behavior for two classes of power grid traffic and then derive three performance measures used in performance analysis: network delay, good foot, and collision rates for two types of traffic. In [33], the authors proposed using wireless multimedia sensors for monitoring purposes in the smart grid. In [34], the authors proposed an iHEM application that uses a wireless sensor home area network (WSHAN) and utilizes communication between the appliance and the energy management device (EMU). In iHEM applications, EMU communicates with appliances, smart meters, and storage devices to determine when it is convenient to accommodate consumers’ needs. The authors compared the performance of the iHEM application with optimization-based scheduling technology. In [35], the authors proposed a distributed algorithm to minimize the data aggregation latency in a smart grid. In [36], the authors studied various routing protocols to provide QoS in a smart grid. In [37], the authors designed a Fi-WSN gateway that prioritizes data, maintains the quality of service (QoS) of Fiber To The Home/Build/Curb (FTTX) users, and delivers WSN data in a reliable manner. The proposed system adopts the Fiber-WSN architecture to support both WSN data and FTTX traffic. In [38], the authors reviewed whether public LTE networks were suitable for smart grid automatic metering and evaluated the same generalized suburban scenarios with hybrid sensors. To achieve more realistic results, the authors modeled the simulation environment with maximum consideration of the impact of the real-world system, including detailed channel properties and topologies. In [39], the authors proposed an Adaptive Wireless Resource Allocation (AWRA) algorithm with QoS guarantee in the communication network of a smart grid. In [40], the authors also presented an overview of Zigbee devices and modules, addressing the importance of smart grid infrastructure and smart metering. The authors provided four case studies to address the most appropriate experimental methodology for implementing Zigbee technology. In [41], the authors proposed a delay response cross-layer scheme with a linear backoff (LDRX) mechanism to address the delay and service requirements of smart-grid-monitoring applications. The LDRX scheme is designed to work in a cluster tree WSN topology suitable for monitoring a wide range of areas, such as substations or large-scale installations. The LDRX scheme is designed to work in a cluster tree WSN topology suitable for monitoring a wide range of areas, such as substations or large-scale installations. The linear backoff period is in any case shorter than the exponential backoff period. Based on this, the node with high priority data exits the backoff period before other nodes. However, if multiple devices try to send data frames with high priority at the same time, it will not be very different from the IEEE 802.15.4 standard. The proposed scheme sets up the backoff interval differently even if multiple devices try to transmit data frames at the same time, and the transmission interval is also distinguished differently. Therefore, the proposed scheme can reduce congestion and delay. In addition, when the number of devices increases, the LDRX scheme increases the probability of collision because the number of allocable backoff intervals in the LDRX scheme is smaller than the exponential backoff. Therefore, in this paper, we propose a new transmission scheme to reduce a delay and performance degradation due to excessive collision in IEEE 802.15.4 for a smart grid.

## 3. Proposed Scheme

**Figure 3:**
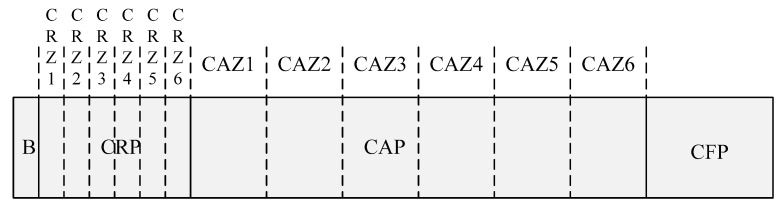
The superframe structure of the proposed scheme.

Figure 3 shows the superframe structure of the proposed scheme.

As shown in Figure 3, the proposed scheme has a contention reservation period (CRP) between the beacon frame (B) and the CAP to reduce the congestion in the CAP, and the devices exchange information with each other in the CRP before entering the CAP period. In the CRP interval, devices exchange information about the amount of packets accumulated in queue so that the transmission attempt is not caught at the same time in the CAP interval. The proposed scheme allows the traffic load in the CAP interval to be distributed naturally according to the information exchanged between the devices in the CRP interval.

The proposed CRP interval has a shorter time slot than the CAP interval, and the length of the CRP can inform devices using the beacon frame including the information on the length of the CRP. In the CRP interval, the devices access the wireless channel using the CSMA/CA mechanism similar to the CAP interval. In the CRP, since the amount of data transmitted by the device is not large, the length of the backoff slot can be set short.

**Figure 4:**
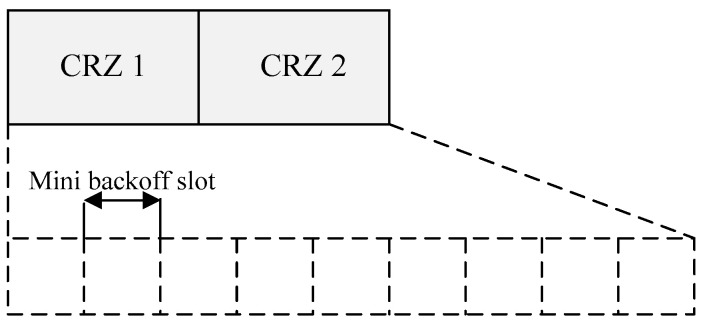
The structure of the proposed CRZ.

In addition, the proposed scheme proposes a contention reservation zone (CRZ) and contention access zone (CAZ) for the load balance. The CRZ denotes each zone that divides the CRP into N zones. Likewise, the CAZ indicates each zone that divides the CAP into N zones. Each CRZ and CAZ correspond one-to-one. For example, when the coordinator sets six CRZs in the CRP and six CAZs in the CAP, CRZ and CAZ are used for load balancing. CRZ n is sequentially mapped to CAZ n. Figure 4 shows the structure of the proposed CRZ.

As shown in Figure 4, the CRZ consists of multiple mini backoff slots. Devices transmit packets using the slotted CSMA/CA operation in the mini backoff slot. The packet transmitted in one CRZ includes queue information of each device, and the device senses the transmission of another device by the slotted CSMA/CA scheme and transmits packets by a backoff algorithm. However, if the number of devices is large, the device may not transmit its packet.

**Figure 5:**
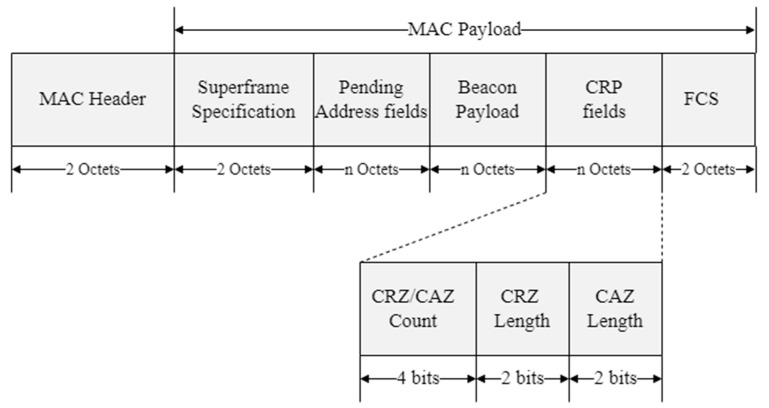
The format of the proposed beacon frame.

When the coordinator expects increases in contention for data transmission in the CAP by backlogged traffic, it broadcasts the beacon frame including the CRP information. The CRP information includes the length of the CRP interval, the number of the CRZ, etc. When the coordinator broadcasting the beacon frame receives the queue information frame from devices, it transmits the ACK frame to the corresponding devices. Figure 5 shows the format of the proposed beacon frame.

The proposed beacon frame includes the MAC header, superframe specification fields, GTS field, pending address fields, and CRP field. The CRZ/CAZ count subfield indicates the number of CRZ/CAZ. The CRZ length subfield indicates the length of one CRZ, and CAZ length subfield indicates the length of one CAZ. If the CRZ/CAZ count subfield is set to 3, the CRZ length subfield is set to 1, and the CAZ length subfield is set to 3, the CRP is 1~3 time slots in the superframe. In addition, the length of one CAZ is 3 time slots, the CAZ interval is from 4th time slot to 12th time slot in the superframe. The remaining time slots 13~15 operate as the CAP.

The coordinator broadcasts the beacon frame including the proposed CRP fields. The device that has received the beacon frame obtains the CRP information and determines the resource amount required for packet transmission, and it determines the number of CAZ based on the amount of resources. The device then arbitrarily selects one or a plurality of CRZs according to the number of CAZs.

The queue information frame includes the length of the remaining queue, the remaining battery life, etc. The coordinator stores CRP information such as interval length and the number of CRZs. The device arbitrarily selects one or a plurality of CRZs according to the number of CAZs detected among the entire CRZ. In order to transmit data frames in the CAP interval, the device first transmits a message including the queue information to the coordinator in the mini backoff slot in the selected CRZ. When an ACK is received from the coordinator, the device decides the CAZ corresponding to the selected CRZ as an exclusive CAP and transmits data frames in the exclusive CAP. If no ACK is received from the coordinator, the device considers that the coordinator has sent a NACK frame and retransmits the queue information message. If the device does not receive the ACK frame from the coordinator in the selected CRZ, it selects one of the remaining CRZs that have not yet been selected. The exclusive CAP means an interval in which data can be transmitted in a larger amount than the amount of data that is basically set.

While the device does not transmit its queue information packet in the selected CRZ, it receives the queue information packet of another device in a listening state. If it receives the ACK frame from the coordinator, it stores the corresponding queue information packet. The stored queue information packet can be used to adjust the CSMA/CA parameters in the CAP interval.

The device only performs listening on the unselected CRZ. If there is no ACK frame for the queue information packet transmitted by another device in the CRZ, the device determines the CAZ corresponding to the CRZ as a normal CAP. The normal CAP means a section for transmitting data frames with an amount of data basically set.

If the device does not transmit the queue information packet in all CRZs, it decides the entire CAP interval as a background CAP. The background CAP means a period for transmitting data in a remaining period of the entire CAP.

The device receives the beacon frame from the coordinator, transmits the queue information packet in each CRZ corresponding to each CAZ, and receives the ACK from the coordinator.

The device stores the queue information packet received from the coordinator for data transmission and stores the CAP information corresponding to the result value according to the transmission of the queue information packet.

**Figure 6:**
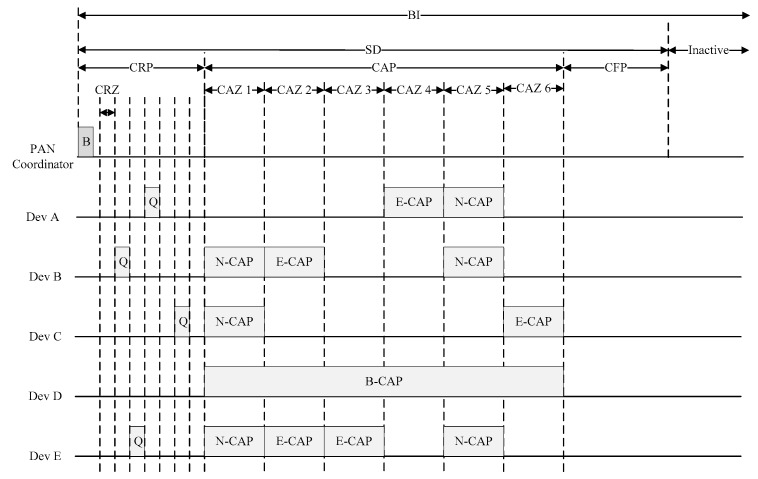
The procedure of data transmission in the proposed scheme.

Figure 6 shows the procedure of the data transmission in the proposed scheme. In a CRZ, when a plurality of devices attempt to transmit queue information packets, all devices may succeed in transmission of the queue information packet, partially succeed, or fail altogether. If all transmission attempts of the queue information are successful, all devices use the exclusive CAP mechanism to access the channel in the corresponding CAP. However, in the case of a large number of devices, it is uncommon for all devices to successfully transmit the queue information packet, and usually only some devices transmit the queue information packet successfully. The device that successfully transmits the queue information packet accesses the channel in the corresponding CAZ using the exclusive CAP mechanism, but the remaining devices cannot transmit data frames in the corresponding CAZ. Devices listening to the channel without the transmission of the queue information packet access the channel using the normal CAP mechanism in the corresponding CAZ. A device that fails to transmit in the CRP interval operates as a background CAP in the entire CAP interval.

In Figure 6, the CAP in the superframe is divided into six CAZs, and the CRP is divided into six CRZs. The coordinator broadcasts a beacon frame including the information on the superframe structure to each device, and each device recognizes the structure of the superframe through the received beacon frame.

First, if devices A and D send the queue information packet to the coordinator in the CRZ 1 in the CRP and do not receive the ACK frame from the coordinator, the remaining devices determine to transmit the data using the normal CAP (N-CAP) mechanism in CAZ 1. When device B transmits the queue information packet to the coordinator in the CRZ 2 and receives an ACK from the coordinator, device B determines to transmit data using an exclusive CAP (E-CAP) mechanism in the CAZ 2.

When the device E transmits the queue information packet to the coordinator in CRZ 3 and receives the ACK frame from the coordinator, it determines to transmit data frame using the E-CAP mechanism in the CAZ 3. When device A transmits the queue information packet to the coordinator in CRZ 4 and receives an ACK from the coordinator, it determines to transmit data using the E-CAP mechanism in the CAZ 4. If devices C and D forward the queue information packet to the coordinator in the CRZ 5 and do not receive the ACK from the coordinator, the remaining devices decide to transmit data frames using the N-CAP mechanism in the CAZ 5. When device C forwards the queue information packet to the coordinator in the CRZ 6 and receives an ACK from the coordinator, it determines to transmit data frames using the E-CAP mechanism in the CAZ 6. Device D, which fails to transmit the queue information packet in all six CRZs, decides to transmit data using a background CAP (B-CAP) mechanism in the CAP.

In the proposed scheme, devices in the CAP interval transmit data frames using each CSMA/CA mechanism defined by each CAP mechanism. In general, the E-CAP mechanism can be configured to attempt to access resources more frequently than the N-CAP mechanism, and the B-CAP can be configured to attempt to access resources more occasionally than the N-CAP mechanism.

**Figure 7:**
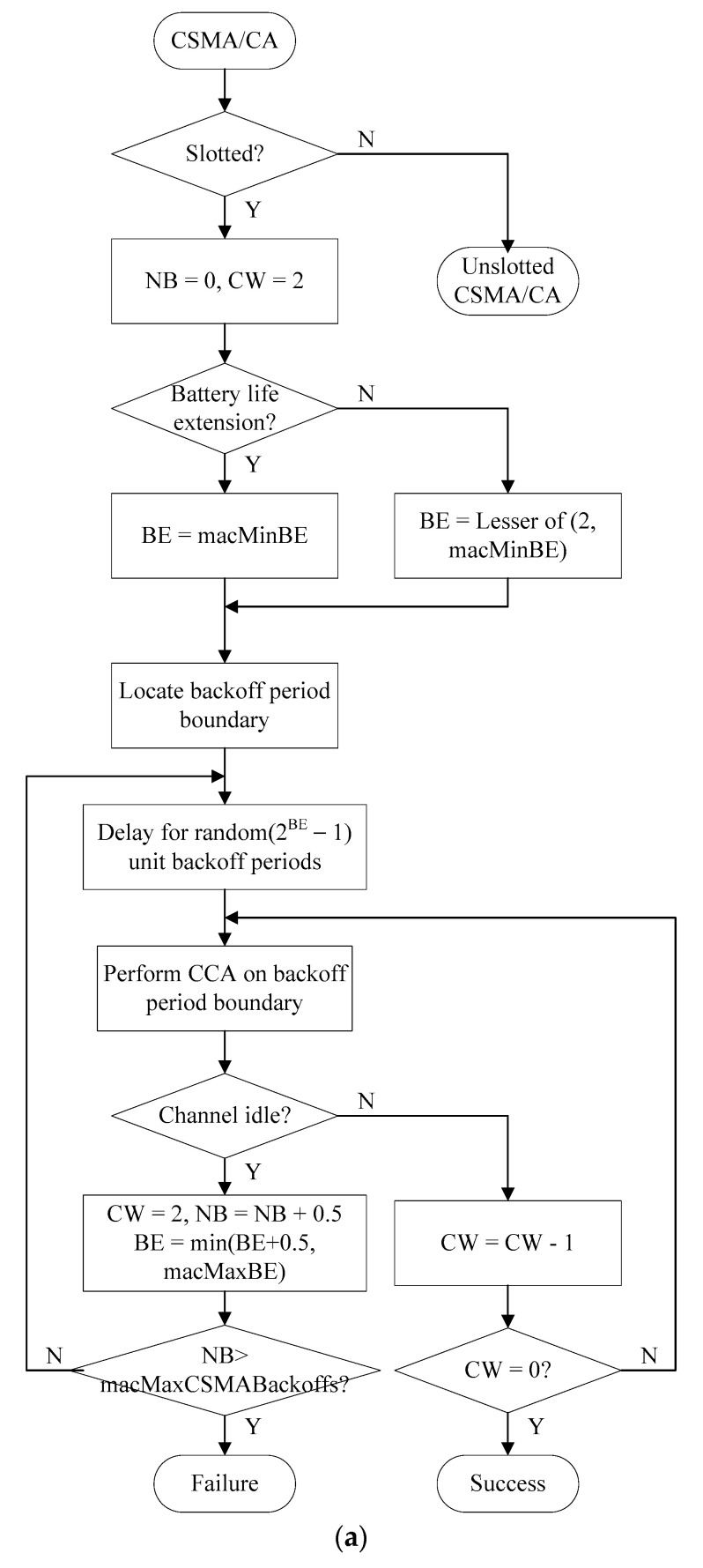

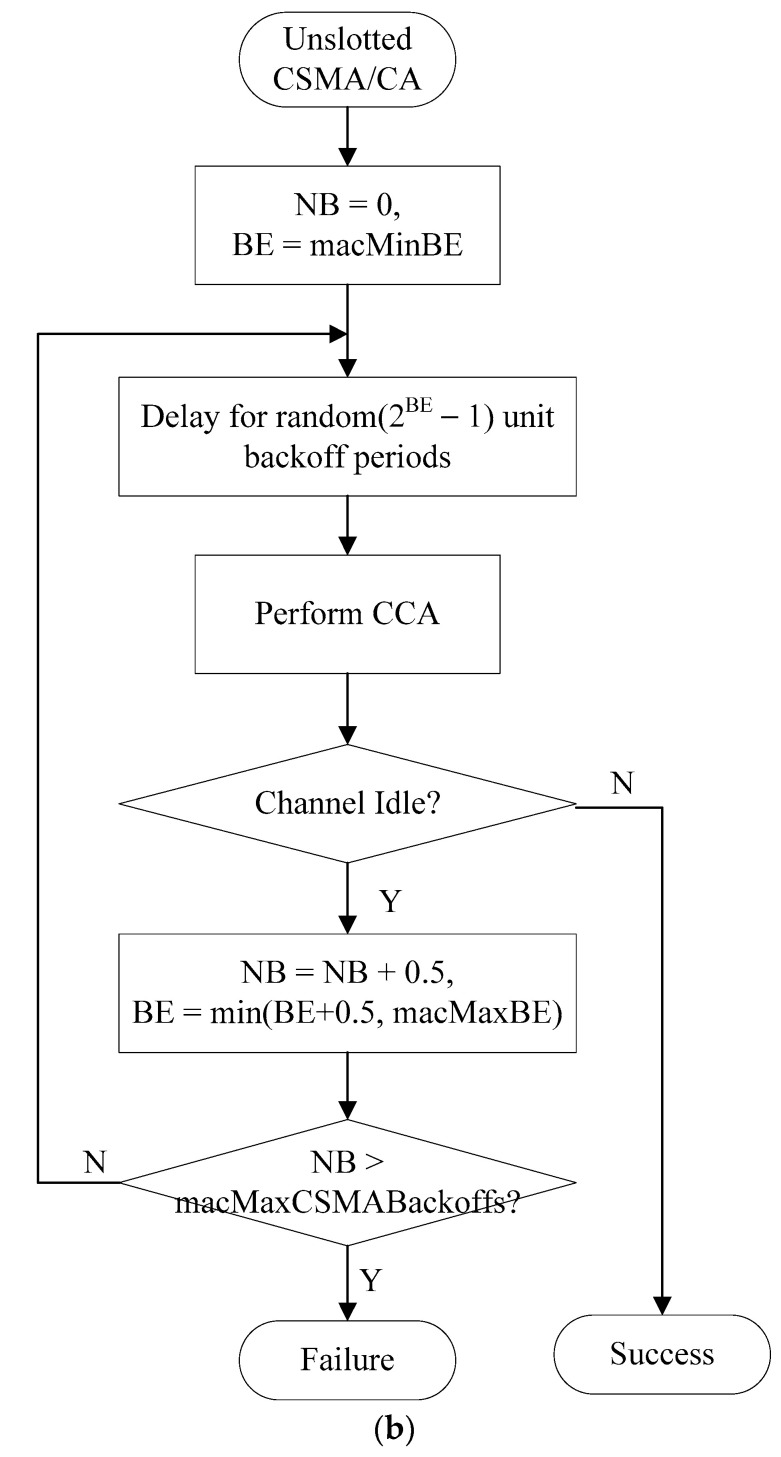
(**a**). The flowchart of the slotted CSMA/CA mechanism in the proposed E-CAP. (**b**). The flowchart of the unslotted CSMA/CA mechanism in the proposed E-CAP.

Figure 7 shows the flowchart of the CSMA/CA mechanism in the proposed E-CAP.

As shown in Figure 7, the E-CAP sets *macMinBE* and *macMaxBE* smaller than the N-CAP, and sets the *BE* increment as follows.

BE=min(BE+0.5, macMaxBE)

In addition, the E-CAP sets the maxCSMAbackoffs value to a large value and sets the *NB* increment as follows.

NB=NB+0.5

The *NB* represents the number of retries due to backoff in one connection attempt. The CW is the number of backoff intervals needed to determine if the channel is idle. The BEs determine the number of backoff periods the device shall wait for before accessing the channel. A device that has a packet ready for transmission first backs off for a random number of backoff slots, chosen uniformly between 0 and 2^BE^−1, before sensing the channel.

The E-CAP sets the *BE* and *NB* less than other CAP schemes and can give devices high channel access. To configure the backoff intervals, the N-CAP uses the *macMinBE*, *macMaxBE*, and maxCSMAbackoffs values defined in the IEEE 802.15.4 standard, and *BE* and *NB* are set as follows.

$$\begin{array}{l} BE=\min\left(BE+1,\;macMaxBE\right)\\ NB=NB+1 \end{array}$$

The CSMA/CA algorithm used in the N-CAP is the same as the CSMA/CA mechanism defined in the IEEE 802.15.4 standard. The B-CAP sets *macMinBE* and *macMaxBE* larger than the N-CAP, and the *BE* for setting the backoff interval is set as follows.

BE=min(BE+2, macMaxBE)

In addition, the B-CAP sets maxCSMAbackoffs small and sets *NB* as follows.

NB=NB+2

The B-CAPs set *NB* and *BE* longer than other CAPs and give devices channel access opportunities lower than other CAP schemes. Therefore, the B-CAP is more likely to access the channel later than other CAP schemes. In addition, in the same CAZ, the proposed scheme can give differentiation connection in consideration of QoS between devices based on queue information exchanged in the CRP section.

**Figure 8:**
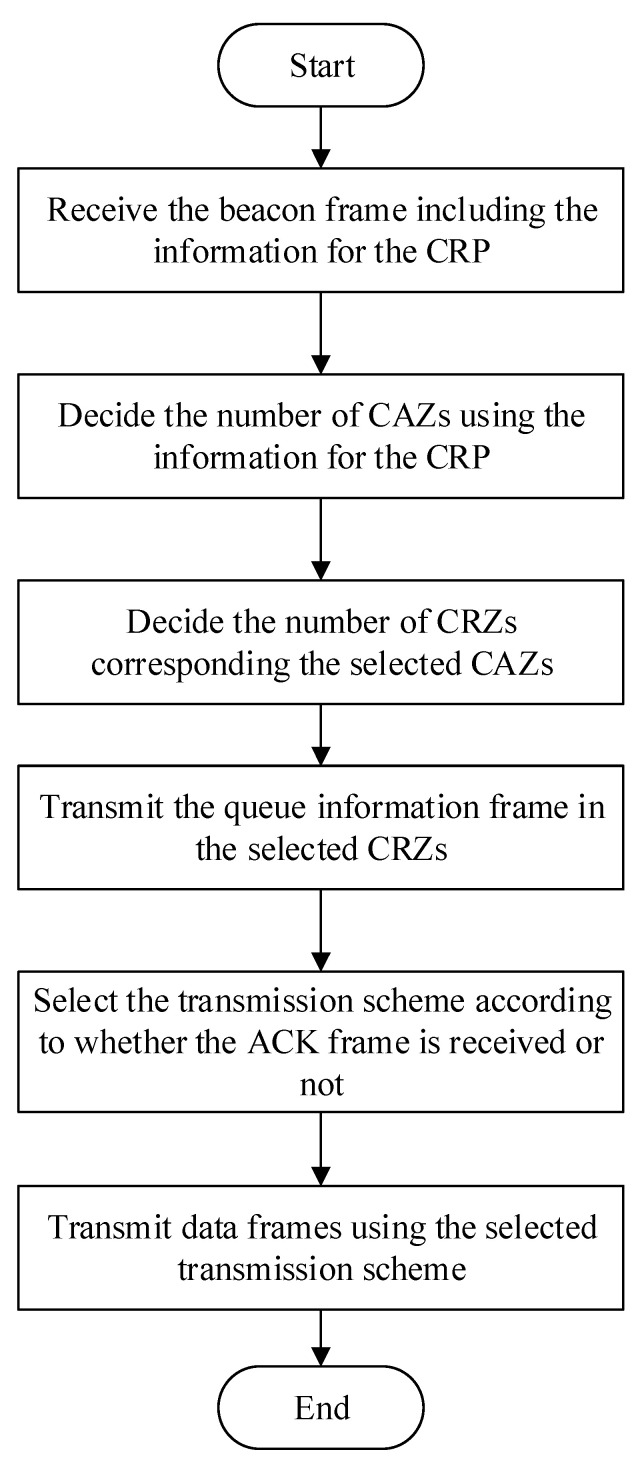
The flowchart of the operation of the device in the proposed scheme.

Figure 8 shows the flowchart of the operation of the device in the proposed scheme. In Figure 8, the device receives the beacon frame containing CRP information from the coordinator. The device identifies the resource requirement for packet transmission based on the received CRP information and determines the number of CAZs according to the identified resource requirement. The device determines the number of CRZs based on the determined number of CAZs and transmits a queue information packet to the coordinator. At this time, the number of CRZs corresponds to the number of CAZs. The device determines the type of CAP according to whether it transmits its queue information packet successfully and whether other devices transmit their queue information packets successfully. For example, when the device receives the ACK frame for the queue information packet from the coordinator, it determines that the transmission of the queue information packet is successful, and it determines the type of the CAP as the E-CAP, and it transmits data frames using the determined E-CAP mechanism. If the ACK frame of the queue information packet transmitted by another device is not detected, the device determines the type of the CAP as the N-CAP and transmits data frames using the determined N-CAP mechanism. On the other hand, if the device fails all of the transmission of the queue information packet in the MBP, it determines the type of the CAP as the B-CAP and transmits data frames using the determined B-CAP mechanism.

In this way, the proposed scheme can perform load balance distributedly for data transmission and enables access to the channel efficiently. Therefore, the proposed scheme can reduce the connection delay and the power consumption, and can provide QoS service to devices.

## 4. Performance Evaluation

**Figure 9:**
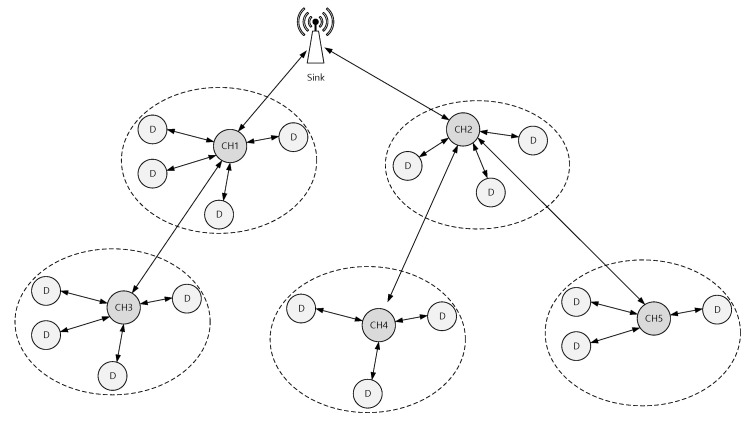
Simulation topology.

We use the QualNet [42] network simulator to simulate the network topology presented in Figure 9 and compare the simulation results with the LDRX schemes [41].

In this simulation, we assume that end devices communicate with their CH using the CAP. We assume that there are a maximum of 20 devices in each cluster, and there are 5 clusters with 5 CHs and a single sink device. In this simulation, we used Poisson traffic arrival. We assume that all of the end devices and CHs operate in the 2.4 GHz band with a bit rate of 250 Kbps. We run each simulation for 400 s and repeat each simulation 10 times.

We assume that all devices within an individual cluster transmit and sense the medium with sufficient power, which means that all devices in a single cluster can hear each other. We also assume that the noise level is constant throughout the entire network. We activate the acknowledgement mechanism to improve the reliability of the system. Table 1 shows some of our simulation parameters; we acquire the rest of the parameters from the IEEE 802.15.4 standard [43] and the energy model of the CC2630, which is a single chip 2.4 GHz IEEE 802.15.4 compliant RF transceiver [44].

Table 2 shows the types of traffic generated by node in the simulation.

The main performance evaluation factors of this simulation were analyzed for packet transmission rate, delay time, and energy consumption. The packet transmission rate is a measure for verifying the reliability of data transmission in a smart grid environment. This represents the ratio of data packets successfully transmitted to the source node, which is successfully transmitted to the destination node. If all packets are received, it is 100%. The delay time represents the average time of data successfully arrived through the multi-hop path. This time is calculated from the time when data are generated to the time when they arrive at the gateway, which is the destination node. Energy consumption is an analysis of the average energy consumption of existing and proposed techniques, confirming that it can guarantee network life in AMI network environments with limited energy. In this simulation, we assume that nodes transmit traffic for AMI and Power Quality Monitoring Application.

**Figure 10:**
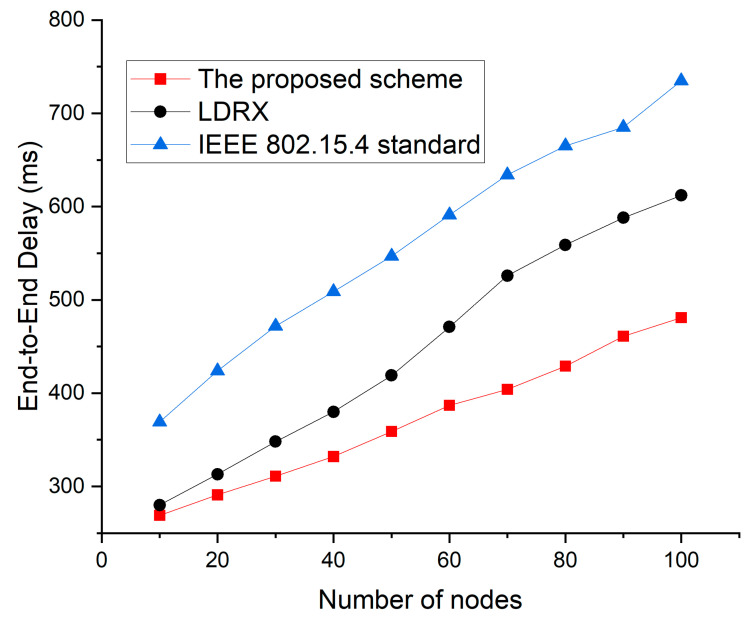
End-to-end delay as a function of the number of devices.

Figure 10 shows the simulation results of the end-to-end delay as a function of the number of devices for the default IEEE 802.15.4 standard, the LDRX scheme, and the proposed scheme.

**Figure 11:**
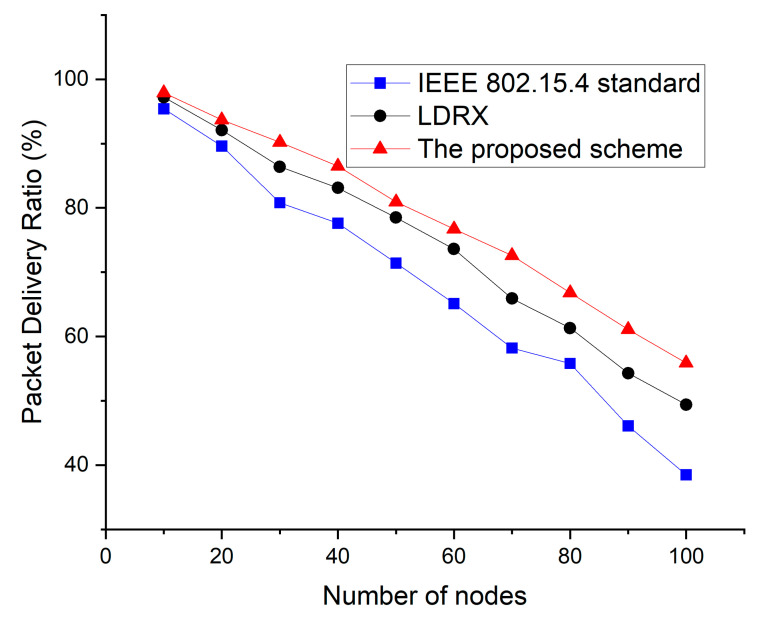
Packet delivery ratio as a function of the number of devices.

In Figure 10, as the number of nodes increases, attempts to transmit data increase greatly, and the collision probability among data frames also increases. Therefore, the overall end-to-end delay also increases significantly. Since the IEEE 802.15.4 standard does not provide a service to reduce competition due to the increase in nodes, the end-to-end delay of the IEEE 802.15.4 standard increases the most. In the case of data frames with higher priority, the LDRX implements a random delay on a period from 0 to (2BE-1) instead of 0 to (2BE-1). Based on this, the node with high priority data exits the backoff period before other nodes. However, when the number of devices increases, the LDRX scheme increases the probability of collision because the number of allocable backoff intervals in the LDRX scheme is smaller than the exponential backoff. Because the proposed scheme sets up the backoff period differently and distinguishes the transmission period differently even if multiple devices attempt data transmission at the same time, the proposed scheme also reduces the end-to-end delay. Figure 11 shows the packet delivery ratio as a function of the number of devices for the default IEEE 802.15.4 standard, the LDRX scheme, and the proposed scheme.

In Figure 11, as the number of devices in the network increases, the number of data transfer attempts increases, and data collisions also increases. Thus, the overall packet delivery ratio is also reduced. The IEEE 802.15.4 standard does not provide a technique to reduce the increase in contention for data transmission when the number of nodes increases. Thus, as the number of nodes increases, the packet delivery ratio of the IEEE 802.15.4 standard is greatly reduced. In the case of LDRX, data frames with higher priority are attempted to be transmitted after a random delay on a period from 0 to (2BE-1) instead of 0 to (2BE-1). Therefore, the packet delivery ratio of the LDRX is higher than that of IEEE 802.15.4 standard because data frames with higher priority have a lower probability of collision than other data frames. However, as the number of devices in the network increases, the transmission of data frames with high priority also increases, and collisions among data frames in the LDRX scheme also increase. Therefore, the packet delivery ratio of the LDRX scheme also decreases significantly. Because the proposed scheme allocates a random delay on the backoff period differently and allocates the transmission intervals differently, it can reduce the competition for data transmission. Thus, the proposed scheme shows better performance than the legacy two schemes.

**Figure 12:**
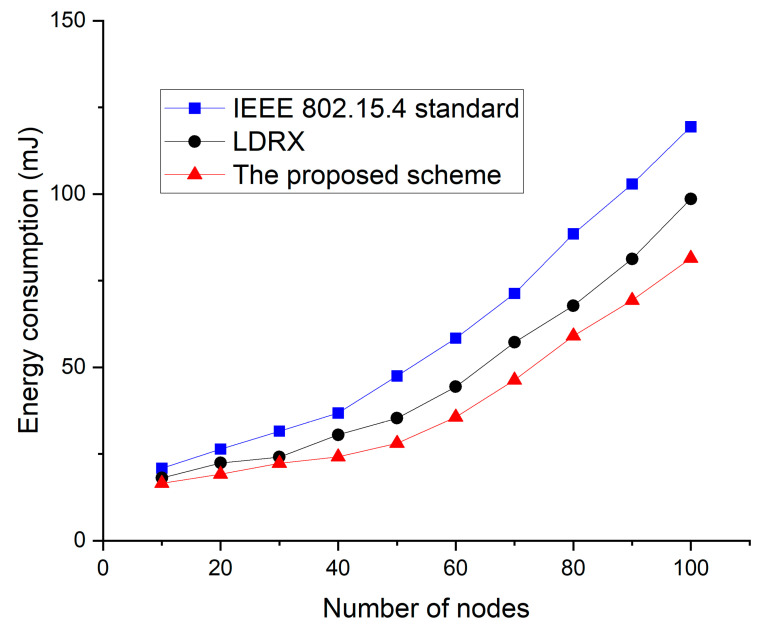
Energy consumption as a function of the number of devices.

Figure 12 shows the energy consumption as a function of the number of devices for the default IEEE 802.15.4 standard, the LDRX scheme, and the proposed scheme. In Figure 12, as the number of devices in the network increases, the data transfer attempts of the devices increase, and the collision probability among data frames also increases. As data collisions increase, the devices attempt to retransmit the data frame, and the energy consumption of the devices also increases. The IEEE 802.15.4 standard does not provide a scheme to reduce the contention for devices to transmit data frames. Thus, the energy consumption of the device in IEEE 802.15.4 standard increases most greatly. Since the LDRX scheme has different backoff allocation intervals according to the priority of data frames, data frames with high priority and data frames with low priority compete for transmission under different conditions. Thus, the LDRX scheme has less competition for data transmission than the IEEE 802.15.4 standard and reduces energy consumption due to retransmission. However, as the number of devices in the network increases, the number of transmissions of data frames with high priority also increases in the LDRX scheme. Therefore, in the LDRX scheme, the collision probability and energy consumption increases. Because the proposed scheme allocates the backoff interval differently and distinguishes the transmission interval of the devices, it can reduce the competition for data transmission more than the legacy two schemes. Therefore, the proposed scheme has less collision probability and energy consumption than the legacy two schemes.

**Figure 13:**
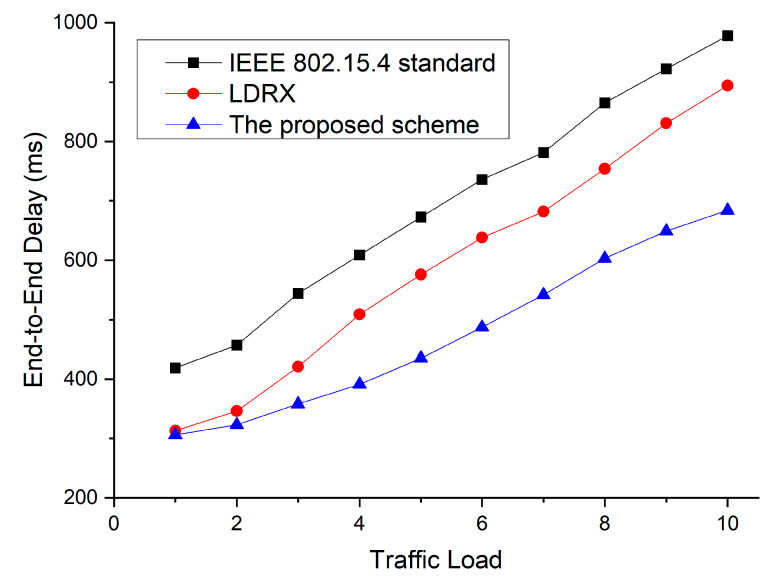
End-to-end delay as a function of traffic load.

Figure 13 shows the end-to-end delay as a function of the traffic load for the default IEEE 802.15.4 standard, the LDRX scheme, and the proposed scheme. In this simulation, we fixed the number of devices in the network to 30. The traffic load refers to the number of times devices in a network transmit data frames within one superframe interval. In Figure 13, as the traffic load increases, the number of data frames sent by devices increases. Therefore, the competition for data transmission increases, and the time required to transfer data also increases. The IEEE 802.15.4 standard does not provide a technique for resolving congestion or a technique for reducing competition. Therefore, the IEEE 802.15.4 standard results in the largest increase in overall end-to-end delay as the traffic load increases. Since the LDRX scheme provides higher data transfer opportunities to data frames with high priority, it can reduce the competition for transmitting data frames with high priority. Thus, the LDRX scheme shows a lower delay than the IEEE 802.15.4 standard. However, as the traffic load increases, the contention among data frames with high priority also increases, and the overall end-to-end delay also increases significantly. Because the proposed scheme distinguishes the backoff interval and the transmission interval according to devices, it provides lower transmission competition than the existing two schemes. Therefore, the proposed scheme shows lower end-to-end delay than the other two schemes.

**Figure 14:**
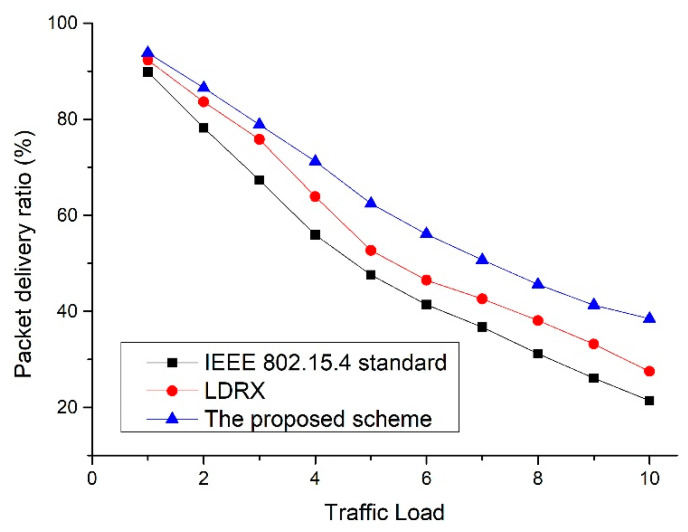
Packet delivery ratio as a function of traffic load.

Figure 14 shows the packet delivery ratio as a function of the traffic load for the default IEEE 802.15.4 standard, the LDRX scheme, and the proposed scheme.

**Figure 15:**
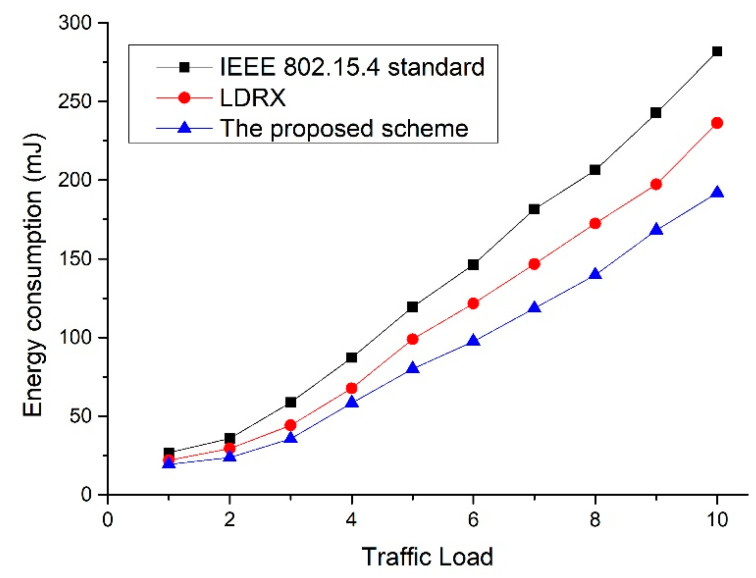
Energy consumption as a function of traffic load.

In Figure 14, as the traffic load increases, the collision probability of the packet increases because the number of data frames that the devices send increases. Thus, the overall packet delivery ratio decreases as the traffic load increases. Since the IEEE 802.15.4 standard does not provide a technique for resolving congestion or competition, the packet delivery ratio is greatly reduced. The LDRX scheme prioritizes data packets to provide higher transmission opportunities for data frames with high priority. Therefore, the packet delivery ratio of the LDRX scheme is higher than that of the IEEE 802.15.4 standard because the data frames with high priority in the LDRX scheme are transmitted in lower contention. However, as the traffic load increases, the number of data frames with high priority increases greatly, and the collision probability among data frames in the LDRX scheme also increases. Therefore, the packet delivery ratio of LDRX decreases significantly as the traffic load increases. The proposed scheme provides a lower competitive environment than the existing two schemes because it gives different backoff and transmission intervals. Therefore, the packet delivery ratio of the proposed scheme is higher than that of the existing two schemes. Figure 15 shows the energy consumption as a function of the traffic load for the default IEEE 802.15.4 standard, the LDRX scheme, and the proposed scheme.

In Figure 15, as the traffic load increases, the number of data frames sent by the devices increases, and the collision probability among data frames increases. Thus, the number of times devices retransmit data frames increases, and the energy consumption by retransmissions also increases. Since the LDRX scheme gives a shorter backoff interval for the transmission of data frames with high priority, data frames with high priority may have more transmission opportunities. Therefore, the LDRX scheme has a low collision probability among data frames in a low traffic load environment, and the number of retransmissions due to the collision is less than that of the IEEE 802.15.4 standard. However, as the traffic load increases, the number of transmissions of data frames with high priority also increases, and the collision probability of data frames also increases. Therefore, the number of retransmissions due to collisions also increases, and the energy consumption of the LDRX scheme also increases significantly. Because the proposed scheme allocates different backoff intervals and transmission intervals depending on the device, it can reduce the competition for data transmission. The data collision probability of the proposed scheme is lower than that of the existing two schemes, and the number of retransmissions due to collisions is also reduced. Therefore, the energy consumption of the proposed technique is lower than that of the existing two schemes.

## 5. Conclusions

The IEEE 802.15.4 standard CSMA/CA with a binary exponential backoff mechanism is not well suited for real-time data transmission. To mitigate the drawback of the IEEE 802.15.4 standard, in this paper, we propose a new transmission scheme to reduce delay and performance degradation due to excessive collision in IEEE 802.15.4 for a smart grid. The proposed scheme can reduce the end-to-end delay and can reduce power consumption by enabling efficient resource access through distributed load control.

Simulation results show the effectiveness of the proposed scheme by reducing the end-to-end delay and by increasing the packet delivery ratio (PDR). The proposed scheme achieves the delay reduction and enhances the performance of the network in monitoring smart grid environments.

## Figures and Tables

**Table 1:** Simulation parameters.

Parameters	Value
Transmission power (dBm)	3.5
Noise factor (dB)	10.0
Synchronization mode	Beacon-enabled
Carrier sense sensitivity	−85 dBm
Contention window	2
IEEE 802.15.4 Header Length	22 bytes
RX current consumption	5.9 mA
TX current consumption	9.1 mA
IDLE current consumption	0.550 mA
Sleep current consumption	0.001 mA
BO	6~10
SO	3~6

**Table 2:** Smart grid application.

Traffic Type	Packet Interval	Packet Size
AMI data	18 s	5 Bytes
SCADA (Supervisory Control and Data Acquisition)	1 s	64 Bytes
Power Quality Monitoring	1 s	35 Bytes
Office Substation	10 s	64 Bytes

## Data Availability

Not applicable.
